# Antibiotic treatment indicates shorter survival in patients with immunotherapy for metastatic kidney cancer

**DOI:** 10.2340/1651-226X.2026.44974

**Published:** 2026-02-10

**Authors:** Elisa Kankkunen, Kaisa Sunela, Timo Makkonen, Katriina Jalkanen, Kalle E Mattila

**Affiliations:** a Department of Urology, Helsinki University Hospital, Helsinki, Finland; b Department of Oncology, Tampere University Hospital, Tampere, Finland; c Comprehensive Cancer Center, Helsinki University Hospital, Helsinki, Finland

**Keywords:** gut microbiome, kidney cancer, prognostic factors, immunotherapy

## Abstract

**Background and purpose:**

Antibiotic treatment (ABT) has been associated with worse outcomes of cancer immunotherapy. However, this association might be confounded by other poor prognostic factors. We aimed to evaluate the use of ABT and outcomes of immune checkpoint inhibitors (ICI) in metastatic kidney cancer (mRCC).

**Patient/material and methods:**

We identified retrospectively 192 patients treated with ICI for mRCC between 2015 and 2021 at three academic hospitals in Finland. Information on patient characteristics, ABT, and immunotherapy was collected from electronic medical records. Cox regression and Kaplan-Meier methods were used for survival analyses.

**Results:**

A total of 61 (32%) patients had received early ABT (3 months before and 1 month after the first dose of ICI), of whom 31 (51%) had ABT > 7 days. Patients with early ABT had shorter median overall survival (mOS) than patients without early ABT (20.4 vs 27.9 months, p = 0.046). Patients with ABT > 7 days had shorter mOS than patients with ABT 0–7 days (17.2 vs 27.5 months, p = 0.015). After adjustment for International Metastatic Renal Cell Carcinoma Database Consortium risk groups, histological renal cell carcinoma subtype, baseline levels of C-reactive protein, and tumor burden, the risk of death was higher in patients with ABT > 7 days (hazard ratio 1.83 (95% confidence interval 1.06–3.17). No significant differences in progression-free survival times (PFS) were observed.

**Interpretation:**

Early ABT and prolonged ABT duration were associated with shorter OS, but not with PFS, in patients treated with ICI for mRCC. Prolonged ABT indicated poor prognosis regardless of other risk factors.

## Introduction

Currently, immune checkpoint inhibitors (ICI) represent the standard treatment option for patients with metastatic clear cell renal cell carcinoma (ccRCC) and have also become an option for patients with metastatic non-ccRCC [1]. Combination therapies with ipilimumab and nivolumab and with PD-1/L1 inhibitors (nivolumab, pembrolizumab, and avelumab) and tyrosine kinase inhibitors (TKIs: cabozantinib, lenvatinib, and axitinib) have outperformed sunitinib in the first-line treatment of metastatic ccRCC, especially in patients with intermediate and poor prognosis [2–5]. Nivolumab has also prolonged progression-free survival (PFS) and overall survival (OS) compared to everolimus in later treatment lines [6]. Immunotherapy has also yielded improved treatment outcomes in real-world patients with renal cell carcinoma (RCC) outside clinical trials [7, 8]. Besides the International Metastatic Renal Cell Carcinoma Database Consortium (IMDC) risk score, there are no predictive biomarkers available to guide the optimal treatment selection for patients with metastatic kidney cancer (mRCC) in routine clinical practice [1].

In addition to a tumor microenvironment, host-related factors, such as gut microbiome, modulate the activity of immune system [9, 10]. Researchers have shown that fecal transplantations affected responses to anti-PD-1 therapy in a preclinical model [11]. It has been proposed that antibiotic treatment (ABT) can hamper the effect of ICI by decreasing the diversity of gut microbiome [10–12], whereas high diversity in fecal microbiome and certain commensal bacteria (*Bifidobacterium longum*, *Collinsella aerofaciens*, *Enterococcus faecium,* and *Bacteroides fragilis*) have improved responses to PD-1 inhibitors in patients with melanoma and mRCC [12–15].

The use of antibiotics has been evaluated in real-world patients who had received ICI for the treatment of several types of cancer. ABT within 30 days prior to PD-1 inhibitors was associated with decreased objective response rates (ORR) and OS in patients with different types of cancer including ccRCC, whereas concurrent use of antibiotics during ICI was not [16]. Early antibiotic use (2 months before and 1 month after the first dose of ICI) was associated with shorter OS in patients with non-small cell lung cancer (NSCLC) as well as with lower ORR and worse PFS in patients with mRCC [17, 18]. ABT 30 days prior to or post the first dose of ICI was associated with shorter PFS and OS in patients with metastatic RCC, NSCLC [19], and melanoma [14]. The duration of ABT might also affect the results of immunotherapy, as patients with cumulative and prolonged courses of ABT had impaired survival compared to patients without antibiotics [20, 21].

In this study, we aimed to evaluate the use of ABT before and during immunotherapy and its effect on treatment outcomes in patients with mRCC in Finland.

## Patients and methods

### Study population

This study included 192 patients who had received at least one dose of nivolumab either as monotherapy or in combination with ipilimumab, cabozantinib, or investigational regimen for mRCC at Helsinki, Turku, and Tampere University Hospitals between 2015 and 2021. Other ICIs were not reimbursed for mRCC in Finland during the study period. Baseline patient and tumor characteristics and information on systemic cancer treatments, disease progression, and death were retrospectively collected from electronic medical records until November 11, 2024. The information on systemic cancer treatments included ICI (nivolumab, ipilimumab, investigational regimen) and TKI (sunitinib, pazopanib, sorafenib, cabozantinib, axitinib, and everolimus). Concurrent treatment with corticosteroids (prednisolone, methylprednisolone, and dexamethasone) during immunotherapy was also collected.

### The assessment of ABTs

All antibiotics were collected from electronic medical records and pharmacy records 90 days before and 30 days after the first dose of nivolumab. The information on ABT included classes (penicillin, tetracyclines, cephalosporins, quinolones, lincomycin, macrolides, sulfonamides, glycopeptides), indications (skin infection, respiratory infection, urinary infection etc.), the route of administration (oral, intravenous, intramuscular), start date, stop date, and the duration and doses of each course of ABT. If the patient had simultaneous courses of ABT, all ABT courses were recorded separately. Early ABT was defined as the use of antibiotics between 3 months before and 1 month after the first dose of ICI.

### Statistical methods

The median follow-up time for study patients was estimated using the reverse Kaplan–Meier method. OS was determined as the time from the first dose of nivolumab to death of any cause. PFS was determined as time from the first dose of nivolumab until disease progression determined by study investigators (EK, KS, TM, KEM) or death. The median OS and PFS estimates with (95% confidence intervals [CI]) were calculated using the Kaplan–Meier method. Statistical significance was assessed using the log-rank test, and all tests were two-sided with a significance threshold of *p* < 0.05. Hazard ratios (HR) with 95% CI for OS and PFS were calculated using multivariable Cox regression analysis. Covariates included (early ABT, duration of ABT, IMDC risk groups, histological RCC subtype, baseline levels of C-reactive protein (CRP), and tumor burden). Analyses were made separately for early ABT and the duration of ABT, due to strong correlation (Cramer’s *V* = 0.64). The proportional hazards assumption was assessed graphically using log-minus-log survival plots. All analyses were performed with SPSS (version 29) and R (version 4.4.1).

## Results

### Patient characteristics

Of 192 mRCC patients treated with ICI during 2015–2021, the median age at the initiation of ICI was 65 years, the majority were male (63%) and had clear cell carcinomas (91%). According to IMDC risk group classification, 37 patients (19%) had a favorable prognosis, 91 (47%) had intermediate prognosis, 56 (29%) had poor prognosis, and the prognostic group could not be determined for 8 (4%) patients. Nivolumab was given as monotherapy to 157 (82%) patients and as combination with ipilimumab to 15 (8%) patients, with cabozantinib to 13 (7%) patients and with investigational/other regimen to 6 (3%) patients. There were 26 (14%) patients who received ICI as first-line treatment, 83 (43%) as the second line treatment, and 83 (43%) as the third or later treatment line. Additionally, 107 (56%) patients received systemic cancer treatments other than ICI: 117 (61%) patients received cabozantinib, 115 (60%) sunitinib, 77 (40%) pazopanib, 62 (32%) everolimus, 60 (31%) axitinib, and 13 (7%) patients sorafenib. Altogether 154 (80%) patients were operated on with radical or partial nephrectomy. A total of 42% of all patients received corticosteroid treatment during ICI. Patient characteristics are described in Table 1.

**Table 1:** Patient characteristics.

Baseline characteristics		*N* = 192 patients
**Sex**	Male	121 (63%)
	Female	71 (37%)
**Age at the initiation of ICI**	Median (years)	65
	Range (years)	22–83
**Baseline IMDC classification**	Favourable	37 (19%)
	Intermediate	91 (47%)
	Poor	56 (29%)
	Missing	8 (4%)
**CRP level at the initiation of ICI**	Normal, ≤ 10 mmol/L	93 (48%)
	Elevated, > 10 mmol/L	94 (49%)
	Missing	5 (3%)
**Histological RCC subtype**	Clear cell RCC	175 (91%)
	Non-clear cell RCC	9 (5%)
	Missing	8 (4%)
**Sarcomatoid differentiation**	Present	15 (8%)
**Tumor burden**	1–2 metastatic sites	89 (46%)
	3 or more metastatic sites	103 (54%)
**Baseline brain metastases**	Present	13 (7%)
**ICI treatment**	Monotherapy	157 (82%)
	Combination therapy	35 (18%)
**ICI treatment line**	1st line	26 (14%)
	2nd line	83 (43%)
	3rd or later line	83 (43%)
**Early ABT**	No early ABT	131 (68%)
	Early ABT	61 (32%)
**Duration of ABT**	ABT 0–7 days	161 (84%)
	ABT > 7 days	31 (16%)

ICI: immune checkpoint inhibitors; CRP: C-reactive protein; RCC: renal cell carcinoma; ABT: antibiotic treatment; Early ABT, antibiotic treatment 3 months before and 1 month after the first dose of nivolumab; IMDC: International Metastatic Renal Cell Carcinoma Database Consortium.

### The use of ABT

**Figure 1:**
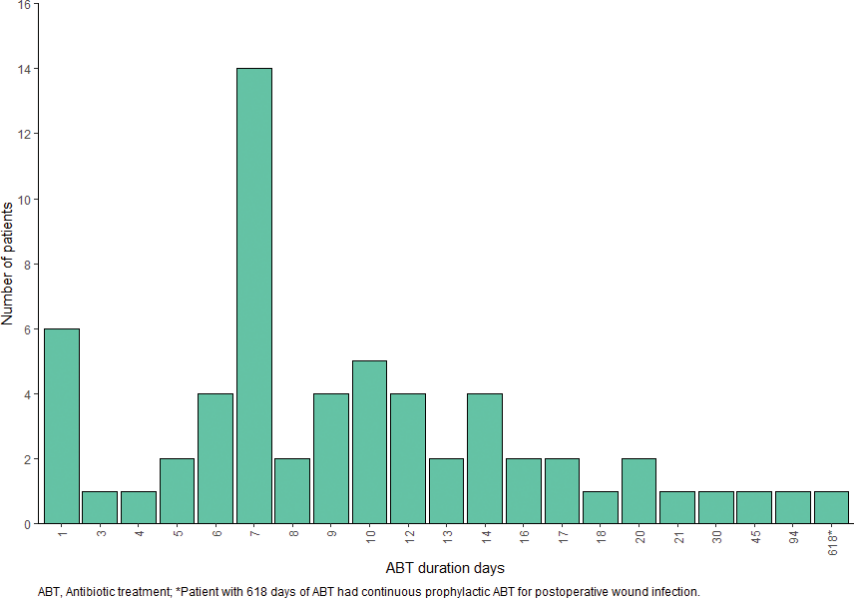
Exposure to ABT.

Early ABT was observed in 61 (32%) patients. Of those, 31 (51%) had ABT > 7 days. Altogether 102 courses of ABT were detected. The duration of exposure to ABT in study patients is illustrated in Figure 1. One course was detected in 33 (54%) patients, two courses in 18 (30%), and three or more courses in 10 (16%) patients. The route of administration was oral in 64 (63%) and intravenous in 38 (37%) of the ABT courses. Cephalosporins, penicillin, and quinolones were the most common classes of ABT used. The most common reason for ABT (43% of all courses of ABT) was respiratory infections, while urinary tract infections caused only 6% of ABT. Unknown infections were reasons for 19% of ABT. There were no patients with sepsis as the indication for ABT in this study population. Classes and indications of ABT are described in Table 2. A total of 57% of patients with early ABT also received corticosteroids during the course of ICI.

**Table 2:** Antibiotic treatments.

Characteristics of ABT		*N* = 102 courses of ABT
**Classes of ABT**	Cephalosporin	45 (44%)
	Penicillin	30 (29%)
	Quinolone	14 (14%)
	Sulphonamide	3 (3%)
	Tetracycline	1 (1%)
	Other	9 (9%)
**Indications of ABT**	Respiratory infections	44 (43%)
	Tooth infections	7 (7%)
	Skin infections	6 (6%)
	Urinary tract infections	6 (6%)
	Abdominal infections	5 (5%)
	Postoperative/wound infections	1 (1%)
	Other infections	14 (14%)
	Unknown infections	19 (19%)
**Route of administration**	Oral	64 (63%)
	Intravenous	38 (37%)

ABT: antibiotic treatment.

### Treatment outcomes

With the median follow-up of 36.87 months (30.80–42.73) from the first dose of nivolumab until censoring or death, 167 (87%) patients had progressed, and 101 (53%) patients had died of any cause. The median OS was 26.83 months (20.89–32.78), and median PFS was 4.23 months (2.69–5.78) for the whole cohort. The ORR was 29% (55 patients). However, 84 (44%) patients had disease progression as the best response. Best response was not assessed or not evaluable in 6 (3%) patients.

### ABT and treatment outcomes

**Figure 2:**
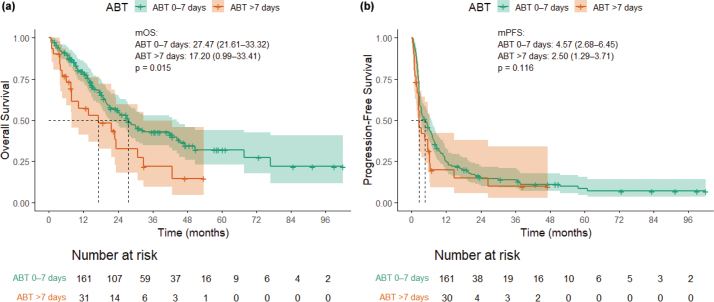
(a) and (b) Overall survival and progression-free survival by the duration of ABT. ABT: antibiotic treatment.

In the univariate analysis, early ABT was associated with shorter OS compared to patients without early ABT (20.37 months (14.58–26.15) vs. 27.90 months (21.25–34.56), *p* = 0.046, HR 1.52 (1.00–2.30)), but not with PFS (2.77 months (0–5.64) vs. 4.47 months (2.53–6.41), *p* = 0.323, HR 1.18 (0.85–1.65)). Longer duration of ABT was also associated with worse OS. Patients with ABT > 7 days had shorter OS compared to patients with ABT duration 0–7 days: 17.20 months (0.99–33.41) vs. 27.47 months (21.61–33.32), *p* = 0.015, HR 1.83 (1.12–2.99). However, the duration of ABT was not significantly associated with shorter PFS (2.50 months (1.29–3.71) vs. 4.57 (2.68–6.45), *p* = 0.116, HR 1.41 (0.92–2.16)). There was no statistically significant difference in the number of cycles of immunotherapy between patients with ABT 0–7 days compared to ABT > 7 days: 74% of patients with ABT 0–7 days have received 1–6 cycles of immunotherapy compared to 62% of patients with ABT > 7 days, *p* = 0.20. In addition, there was no statistically significant difference in ORR between patients with ABT 0–7 days (31%) compared to patients with ABT > 7 days (24%), *p* = 0.49. Differences in OS and PFS are illustrated in Figure 2a and 1b. Univariate analyses for OS and PFS are described in Supplementary Table 1.

### Multivariate analysis of prognostic factors

In the multivariate Cox regression analysis adjusted for IMDC risk groups, histological RCC subtype, baseline levels of CRP, and tumor burden, the duration of ABT > 7 days was independently associated with shorter OS (HR of death 1.83 (95% CI 1.06–3.17)) compared to patients with ABT duration 0–7 days, Table 3. However, longer duration of ABT was not significantly associated with short PFS in multivariate analysis.

**Table 3:** Multivariate cox models for OS and PFS.

	Overall survival		Progression-free survival	
	HR	95% CI	HR	95% CI
**Duration of ABT**				
ABT 0–7 days	Ref.		Ref.	
ABT > 7 days	1.83	1.06*–*3.17*	1.53	0.95*–*2.46
**Baseline IMDC risk group**				
Favorable	Ref		Ref	
Intermediate	0.94	0.52*–*1.71	0.58	0.38*–*0.89
Poor	1.76	0.92*–*3.35	0.86	0.53*–*1.41
**Histological subtype**				
Clear cell	Ref.		Ref.	
non-clear cell	2.26	0.90*–*5.69	2.12	1.03*–*4.36*
**CRP level at the initiation of ICI**				
≤ 10 mmol/L	Ref.		Ref.	
> 10 mmol/L	2.05	1.28*–*3.28*	1.17	0.81*–*1.69
**Tumor burden**				
1–2 sites	Ref.		Ref.	
3 or more	1.61	1.02*–*1.52*	1.22	0.87*–*1.71

ABT: antibiotic treatment; Early ABT: antibiotic treatment 90 days before and 30 days after the first dose of ICI; IMDC: International Metastatic Renal Cell Carcinoma Database Consortium; ICI: immune checkpoint inhibitor; HR: hazard ratio; CI: confidence interval; OS: overall survival; PFS: progression-free survival.

* Statistically significant association.

## Discussion and conclusion

Previous studies have shown the potential effect of gut microbiome on the efficacy of immunotherapy [13, 19, 22–24] and the association of ABT with poor outcomes in various cancers treated with ICI [25, 26]. In this study, we observed that exposure to early ABT and the duration of ABT over 7 days were associated with worse OS in patients treated with ICI for mRCC.

Our findings support evidence on ABT as an indicator of poor prognosis in patients who have received ICI for metastatic cancer. In this study, 32% of patients with mRCC received ABT 3 months before to 1 month after the first dose of ICI which is similar to the rate of early ABT in patients with ICI for NSCLC and melanoma [24]. ABT might be also prescribed for signs and symptoms related to cancer progression. Unknown infections have been reported even in 44% of patients with cancer [25]. In this study, 19% of patients received ABT for unknown infections which was the second most common reason for ABT after respiratory infections. Moreover, 51% of patients with early ABT had been exposed to ABT for more than 7 days which was independently associated with a higher risk of death regardless of IMDC risk groups, histological RCC subtype, baseline levels of CRP, and tumor burden. However, as early ABT and longer duration of ABT were not significantly associated with PFS gained with ICI, the direct effect of ABT on the efficacy of immune checkpoint blockade remains unclear. Moreover, PFS might be a suboptimal surrogate for long-term benefits of immunotherapy. In the CheckMate 214 study with first-line ipilimumab and nivolumab versus sunitinib in patients with metastatic clear cell RCC, there was no statistically significant difference in the PFS between treatment groups despite higher rate of long-term survivors with ipilimumab and nivolumab leading to a statistically significant difference in the OS [2].

The limitations to our study are attributed to its retrospective design. Currently, PD-1/L1 inhibitors are typically used upfront as the first-line treatment for patients with mRCC. In this study cohort, nivolumab was mostly used as monotherapy in second or later treatment lines reflecting the indications of immunotherapy during earlier years of this study. We analyzed the association between ICI treatment line and ORR and observed a statistically significant association. This association is expected, as outcomes generally get worse with later treatment lines due to treatment resistance and disease progression. We did not find significant difference between ABT 0-7 days and > 7 days and the patients treated first line (13% vs 16%), second line (44% vs 39%), third or later line (43% vs 45%), respectively. The groups were comparable and therefore it is unlikely for the treatment line to have a major impact on the survival difference between ABT groups.

The information on ICI and ABT administered at each study hospital was comprehensively collected from hospital medical records. Antibiotics prescribed outside hospital visits were manually searched from national pharmacy records. Although there are electronic prescriptions in Finland, it is possible that we might have missed some prescriptions. In our analysis, ABT duration was calculated as total number of administered days with some overlapping courses of different ABT. There was also lack of information on dietary habits, use of probiotics, and living in rural or urban environments, which could affect gut microbiome in addition to ABT [27] and potentially affect the results of our study. It was common (57%) that patients with early ABT had also received corticosteroid treatment during ICI. Although there is conflicting evidence on the effect of corticosteroid treatment on outcomes of ICI [28], there is evidence that corticosteroid treatment impairs survival in patients with mRCC treated with ICI [29] and this could have affected our results warranting further research in patients with mRCC. Prospective sequential stool and blood sampling from patients treated with immunotherapy are warranted in future studies to characterize the effects of ABT on the immune system and verify the results of retrospective studies.

In conclusion, exposure to early ABT indicates shorter OS in patients treated with immunotherapy for mRCC. As the duration of ABT over 1 week was independently associated with an increased risk of death after adjusted for IMDC risk groups, histological RCC subtype, baseline levels of C-reactive protein, and tumor burden, long courses of ABT should be carefully considered in patients who are candidates for immunotherapy.

## Supplementary Material



## Data Availability

At present, the Finnish legislation on secondary uses of healthcare data and patient records does not permit sharing this type of data. Therefore, the datasets generated and used for this study cannot be made available. Documentation on data-collection processes and dataset are available from the corresponding author on reasonable request.
