# Supplementary material for: Antibiotic treatment indicates shorter survival in patients with immunotherapy for metastatic kidney cancer
Source: Acta Oncol. 2026 Feb 10;65:44974. doi: 10.2340/1651-226X.2026.44974 (PMC12902910; doi:10.2340/1651-226X.2026.44974)
Supplement: Supplementary file 1 [file AO-65-44974-s1.pdf]

Supplementary material has been published as submitted. It has not been copyedited, or typeset by Acta Oncologica

Table 3a) Univariate analyses for OS

		**Overall survival**	
		**Median (95 % CI)**	**HR (95 % CI)**
**Duration of ABT**	*ABT 0-7 days*	27.47 (21.61–33.32)	Ref.
	ABT > 7 days	17.20 (0.99–33.41)	1.83 (1.12–2.99) *
**Early ABT**	*No Early ABT*	27.90 (21.25–34.56)	Ref.
	Early ABT	20.37 (14.58–26.15)	1.52 (1.00–2.30)
**Sex**	*Male*	27.47 (21.78–33.15)	Ref.
	Female	21.63 (15.23–28.03)	1.09 (0.73–1.64)
**Age**	*≤ 65 years*	24.33 (17.55–31.11)	Ref.
	> 65 years	28.53 (18.34–38.73)	0.92 (0.62–1.36)
**Baseline IMDC risk group**	*Favourable*	31.47 (2.30–60.64)	Ref
	Intermediate	30.80 (12.92–48.68)	1.19 (0.68–2.10)
	Poor	15.40 (9.51–21.29)	2.50 (1.39–4.51) *
**CRP level at the initiation of ICI**	*≤10mmol/L*	42.87 (28.36-57.37)	Ref.
	>10mmol/L	14.67 (11.94-17.39)	2.64 (1.75-3.96) *
**Histological RCC subtype**	*Clear cell*	27.90 (22.36–33.44)	Ref
	Non-clear cell	12.57 (5.32–19.81)	2.04 (0.89–4.68)
**Tumor burden**	*1–2 metastatic sites*	41.73 (22.71–60.76)	Ref.
	≥ 3 metastatic sites	19.40 (15.50–23.30)	2.00 (1.32–3.02) *

ABT, antibiotic treatment; Early ABT, antibiotic treatment 90 days before and 30 days after the first dose of ICI; IMDC, International Metastatic Renal Cell Carcinoma Database Consortium; ICI, immune checkpoint inhibitor; HR, hazard ratio; CI, confidence interval; *statistically significant association

Table 3b) Univariate analyses for PFS

		Progression-free survival	
		**Median (95 % CI)**	**HR (95 % CI)**
**Duration of ABT**	*ABT 0-7 days*	4.57 (2.68–6.45)	Ref.
	ABT >7 days	2.50 (1.29–3.71)	1.41 (0.92–2.16)
**Early ABT**	*No ABT*	4.47 (2.53–6.41)	Ref.
	Early ABT	2.77 (0–5.64)	1.18 (0.85–1.65)
**Sex**	*Male*	3.47 (1.90–5.04)	Ref.
	Female	5.33 (2.78–7.79)	0.86 (0.62–1.19)
**Age**	*≤ 65 years*	3.37 (1.83–4.91)	Ref.
	> 65 years	5.33 (3.29–7.38)	0.94 (0.69–1.27)
**Baseline IMDC risk group**	*Favourable*	3.13 (0.59–5.68)	Ref
	Intermediate	4.90 (2.38–7.42)	0.71 (0.47–1.06)
	Poor	3.33 (0.59–6.07)	1.03 (0.67–1.59)
**CRP level at the initiation of ICI**	*≤10mmol/L*	6.43 (4.69-8.17)	Ref.
	>10mmol/L	2.53 (2.19-2.88)	1.42 (1.04-1.93) *
**Histological RCC subtype**	*Clear cell*	4.57 (2.71–6.42)	Ref
	Non-clear cell	2.50 (1.19–3.82)	2.00 (1.02–3.93) *
**Tumor burden**	*1–2 metastatic sites*	5.65 (2.63–8.63)	Ref.
	≥ 3 metastatic sites	3.33 (1.83–4.84)	1.35 (0.99–1.83)

ABT, antibiotic treatment, Early ABT, antibiotic treatment 90 days prior to 30 days after ICI initiation, IMDC, International Metastatic Database Consortium; HR, hazard ratio; CI, confidence interval; *statistically significant association
